# Supplementary figures and images for: High salt diet impairs dermal tissue remodeling in a mouse model of IMQ induced dermatitis
Source: PLoS One. 2021 Nov 1;16(11):e0258502. doi: 10.1371/journal.pone.0258502 (PMC8559960; doi:10.1371/journal.pone.0258502)

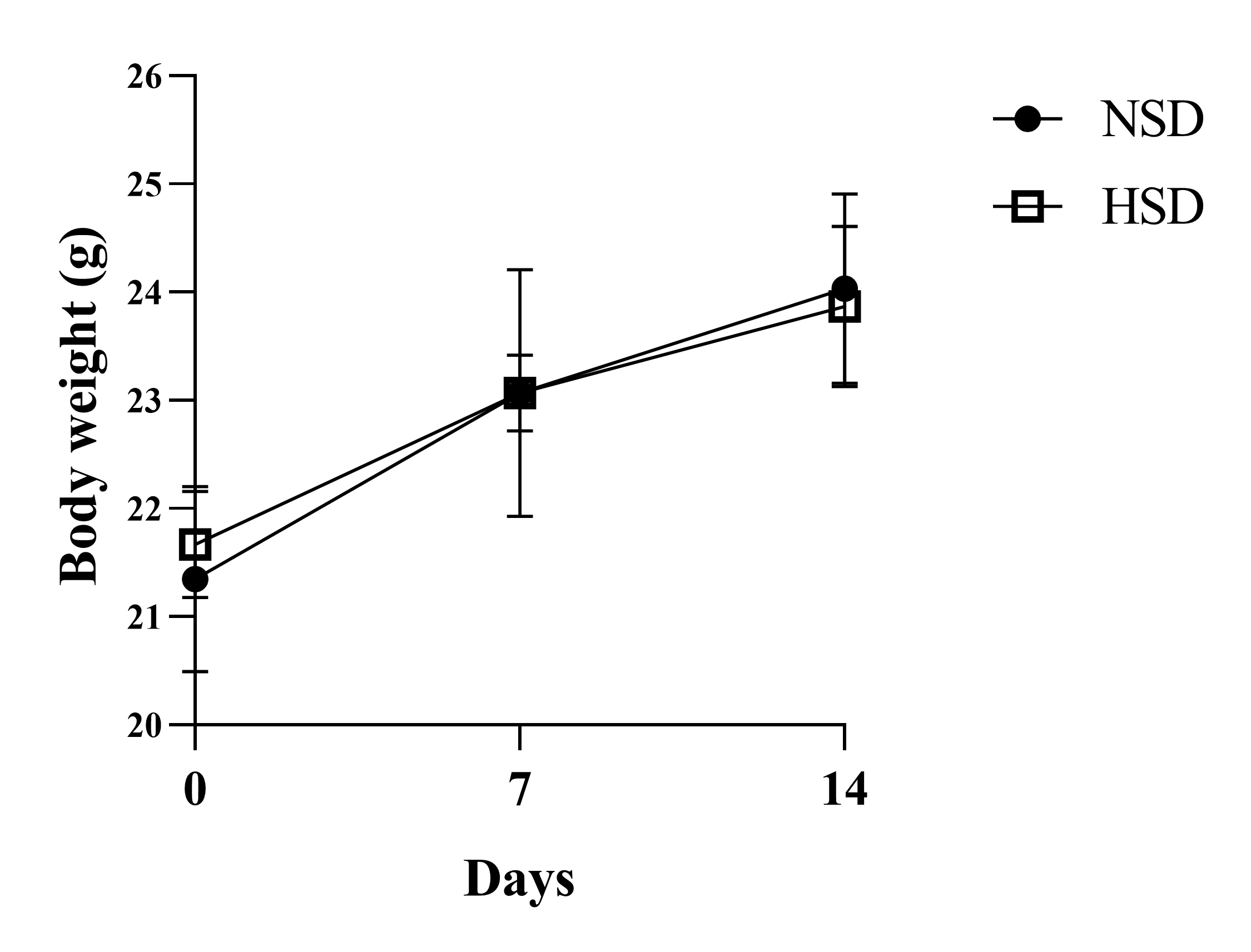

Supplement: S1 Fig — Mice were kept on NSD or HSD for 14 days. The body weight was measured in every 7 days for two weeks. (n = 6 in each group). (TIF) [file pone.0258502.s001.tif]

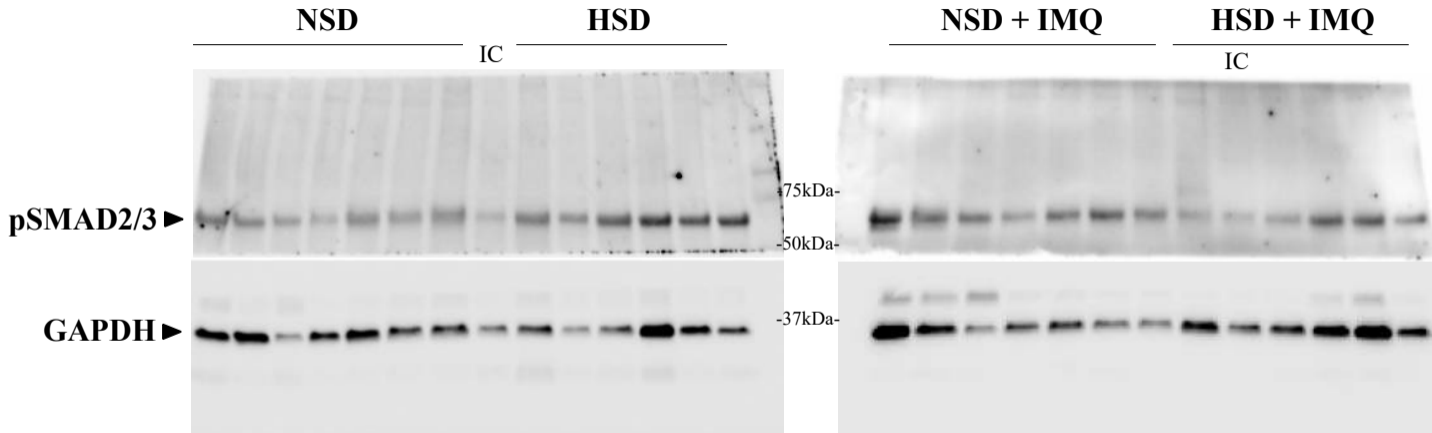

Supplement: S1 Raw images — Internal control (IC). (PDF) [file pone.0258502.s002.pdf]
